# Neoepitopes at the crossroads of immunometabolism: metabolic remodeling of antigen presentation in type 1 diabetes

**DOI:** 10.3389/fimmu.2026.1744422

**Published:** 2026-01-30

**Authors:** Rahul Mittal, Rebecca Goldmann, Mannat Mittal, Naisha Chaudhary, Vibha Ravindra, Khemraj Hirani

**Affiliations:** 1 Diabetes Research Institute, University of Miami Miller School of Medicine, Miami, FL, United States; 2 Division of Endocrinology, Diabetes, and Metabolism, Department of Medicine, University of Miami Miller School of Medicine, Miami, FL, United States

**Keywords:** immune tolerance, immunometabolism, major histocompatibility complex class I, metabolic reprogramming, neoepitopes, redox signaling, type 1 diabetes, β-cell stress

## Abstract

Type 1 diabetes (T1D) is an autoimmune disorder driven by progressive destruction of pancreatic β-cells under conditions of metabolic and oxidative stress. This article examines the intersection of immunometabolism and antigen presentation as a central mechanism in T1D pathogenesis. In β-cells, endoplasmic reticulum (ER) stress, mitochondrial dysfunction, and redox imbalance remodel the immunopeptidome, promoting neoepitope formation and upregulation of major histocompatibility complex class I (MHC-I) molecules. Concurrently, antigen-presenting cells (APCs) exposed to hypoxia, cytokines, and nutrient deprivation undergo metabolic reprogramming that enhances glycolysis, reactive oxygen species (ROS) production, and pro-inflammatory antigen processing. These parallel responses establish a self-sustaining β-cell–APC loop in which metabolic distress in one cell type amplifies dysfunction in the other. By integrating evidence from redox signaling, immunopeptidomics, and metabolic regulation, this perspective defines a unified framework wherein metabolism acts as both initiator and amplifier of autoimmunity. Targeting the immunometabolic interface between β-cells and APCs may restore immune tolerance and prevent disease progression by re-establishing cellular homeostasis.

## 1 Introduction

Type 1 diabetes (T1D) is a chronic autoimmune disease characterized by the targeted destruction of insulin-producing β-cells within the pancreatic islets (1–5). Classically, this process has been attributed to autoreactive cytotoxic CD8^+^ T lymphocytes that recognize β-cell–derived peptides presented by major histocompatibility complex class I (MHC-I) molecules (6). Although immune cells are the immediate effectors of β-cell death, increasing evidence suggests that β-cells themselves are active participants in the autoimmune process (7, 8). Rather than being inert targets, they exhibit a dynamic stress response to metabolic, inflammatory, and viral stimuli that profoundly alters their phenotype and immunogenicity (9).

### 1.1 β-Cells as active participants in autoimmune initiation

**Figure 1:**
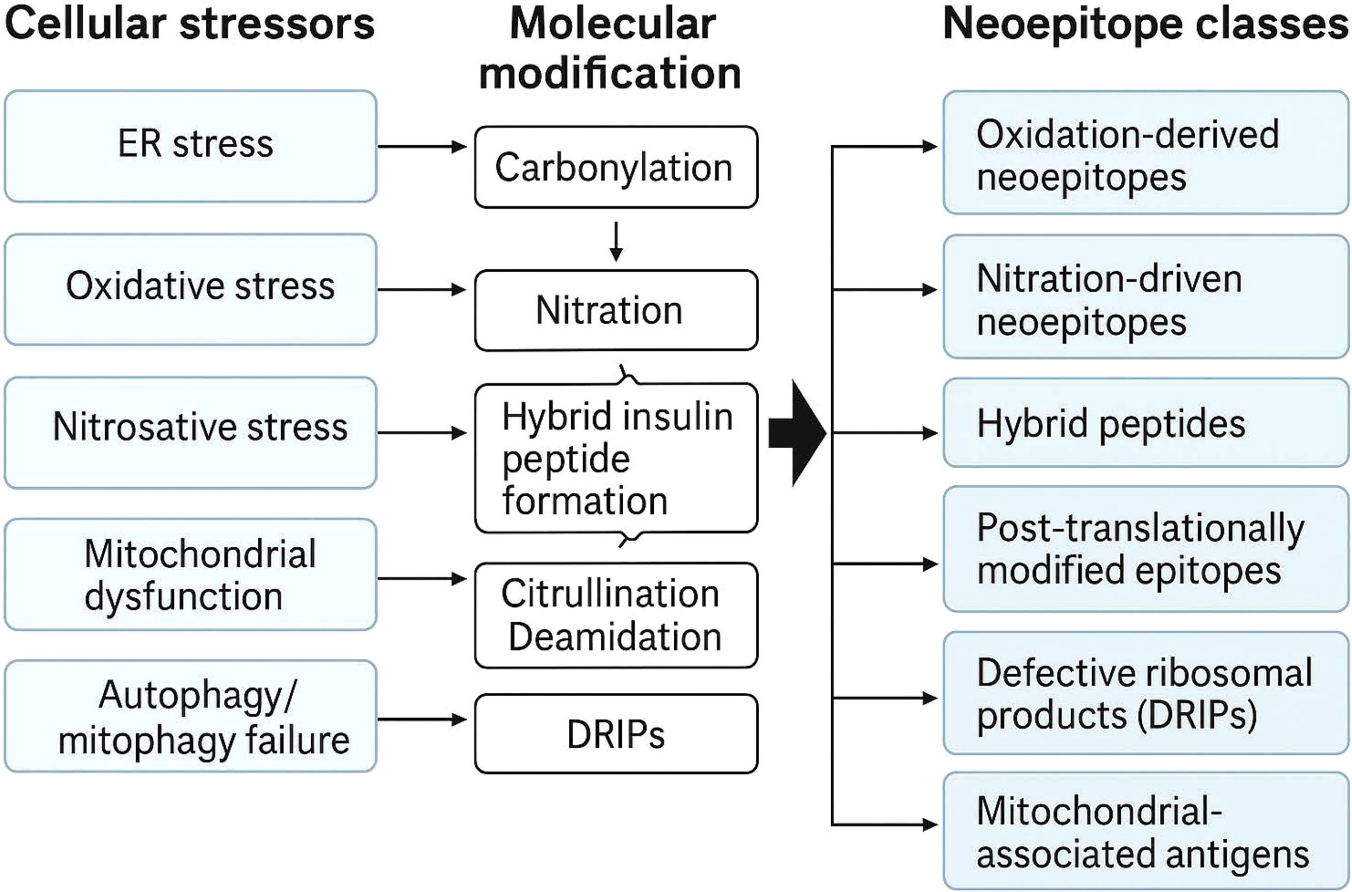
Cellular stress–induced molecular modifications generating neoepitope classes in pancreatic β-cells. ER stress, oxidative and nitrosative stress, mitochondrial dysfunction, and impaired autophagy induce biochemical modifications such as carbonylation, nitration, hybrid insulin peptide formation, citrullination, deamidation, and defective ribosomal product generation. These processes generate distinct neoepitope classes that remodel the β-cell immunopeptidome and enhance immunogenicity.

Under conditions of metabolic or inflammatory stress, β-cells activate adaptive pathways that alter both cellular homeostasis and antigen presentation (10). The secretory burden of insulin biosynthesis renders β-cells especially vulnerable to endoplasmic reticulum (ER) stress, and their relatively limited antioxidant defense predisposes them to oxidative injury (11). These stress responses activate the unfolded-protein response (UPR), mitochondrial stress signaling, and redox-sensitive transcriptional programs that modify peptide processing and antigen presentation. As a result, the β-cell surface repertoire of MHC-bound peptides changes both qualitatively and quantitatively, generating neoepitopes that can be recognized by autoreactive T cells (**Figure 1**). Autoimmune diseases often arise when immune surveillance mechanisms begin to recognize previously hidden or structurally altered self-antigens (12).

### 1.2 Immunometabolism: a framework linking metabolism to immune recognition

The concept of immunometabolism provides a unifying framework for understanding these processes (13, 14). Immunometabolism describes how cellular metabolic pathways control immune-cell differentiation, activation, and effector function (15). Emerging evidence suggests that metabolic intermediates act not only as energy sources, but also as signaling molecules that influence transcription, translation, and epigenetic modification. In the context of T1D, both immune and non-immune cells operate within a shared metabolic network in which alterations in glucose, lipid, and amino acid metabolism reshape immune recognition.

### 1.3 Metabolic regulation of antigen-presenting cells

Within this framework, antigen-presenting cells (APCs) such as dendritic cells, macrophages, and B cells also undergo profound metabolic adaptations in response to environmental cues (16). Their ability to process and present antigens is tightly regulated by nutrient-sensing pathways, including mechanistic target of rapamycin complex 1 (mTORC1), AMP-activated protein kinase (AMPK), and the integrated stress response mediated by the general control non-derepressible 2 (GCN2) kinase (17, 18). Activation of glycolytic metabolism enhances pro-inflammatory cytokine production and co-stimulatory molecule expression, whereas reliance on oxidative phosphorylation is often associated with tolerogenic or anti-inflammatory phenotypes (19–22). These distinct metabolic states determine whether an APC promotes immune tolerance or triggers effector T-cell activation.

### 1.4 A unifying hypothesis: β-cell and immune-cell metabolic stress as a shared axis of disease

The hypothesis advanced in this perspective is that metabolic stress within β-cells and APCs constitutes a unifying axis that underpins the initiation and propagation of T1D. In β-cells, ER stress, mitochondrial dysfunction, and oxidative imbalance remodel the antigenic landscape through altered peptide processing and post-translational modifications (PTMs) (10). These processes give rise to modified self-peptides and hybrid epitopes that are efficiently presented on upregulated MHC-I molecules. Simultaneously, APCs exposed to the metabolic and inflammatory milieu of the islet adapt their bioenergetic programs in a manner that enhances antigen uptake, processing, and presentation. Nutrient deprivation, hypoxia, and cytokine signaling converge on metabolic checkpoints that bias APCs toward an immunogenic presentation mode (23).

### 1.5 Establishment of a feed-forward β-cell–APC immunometabolic loop

The interaction between these two stressed cell populations generates a feed-forward loop of autoimmunity. Metabolically stressed β-cells release danger-associated molecular patterns and neoantigens that are captured and presented by metabolically reprogrammed APCs. In turn, activated APCs secrete pro-inflammatory cytokines such as type I interferons (IFN), tumor necrosis factor (TNF), and interleukin-1β (IL-1β), which exacerbate β-cell stress, increase MHC-I expression on β-cells, and promote further antigen generation and immune activation. This reciprocal amplification may begin years before the onset of hyperglycemia and could represent the earliest cellular event in autoimmune diabetes.

### 1.6 Reframing T1D as a disorder of cellular homeostasis

Understanding T1D through the lens of immunometabolism therefore reframes the disease as a disorder of cellular homeostasis rather than as a purely immune-mediated attack. The β-cell and the immune system are metabolically intertwined, and the disruption of this metabolic dialogue contributes to the failure of immune tolerance. By elucidating the molecular mechanisms linking metabolism, redox signaling, and antigen presentation, new therapeutic strategies can be envisioned that restore β-cell resilience and recalibrate APC function before irreversible β-cell loss occurs.

## 2 β-cell metabolic stress and antigenicity

The pancreatic β-cell is a highly specialized endocrine cell that maintains glucose homeostasis through rapid and tightly regulated secretion of insulin (24, 25). This demanding function requires continuous synthesis, folding, and trafficking of insulin molecules and depends on finely balanced mitochondrial and ER activity. As the β-cell possesses limited antioxidant defenses and a relatively small capacity for protein quality control, it is exceptionally vulnerable to metabolic perturbation. Conditions such as nutrient excess, viral infection, and local inflammation easily disturb its homeostasis, generating stress responses that impair insulin secretion and alter the antigenic characteristics of the cell surface. These metabolic disturbances produce reactive oxygen and nitrogen species, perturb redox signaling, and promote organelle dysfunction. The combined effect is an increase in both β-cell fragility and immune visibility (**Table 1**).

**Table 1:** Metabolic stress pathways in β-cells and their impact on antigen presentation.

Mechanism/Stress pathway	Molecular events	Immunologic consequences	Key references
Endoplasmic Reticulum (ER) Stress	Activation of PERK, IRE1α, and ATF6 pathways; XBP1 splicing; CHOP and JNK activation	Upregulation of MHC-I, TAP1/2, β2-microglobulin; enhanced antigen presentation	(32–37)
Mitochondrial Dysfunction	Impaired oxidative phosphorylation; ROS generation; mtDNA and cardiolipin release	Activation of cGAS–STING pathway, type I IFN signaling, neoepitope formation	(45–51)
Redox Imbalance/Oxidative Stress	Protein carbonylation, nitration, and hybrid peptide formation	Creation of neoepitopes recognized by autoreactive T cells	(38, 42–44, 53)
HLA Class I Hyperexpression	IFN-α/β–driven JAK–STAT signaling; immunoproteasome formation	Increased β-cell visibility to cytotoxic CD8^+^ T cells	(6, 76–79, 81, 82)
Autophagy/Mitophagy Defects	Impaired clearance of damaged mitochondria and misfolded proteins	Amplified ER stress, antigen accumulation, MHC-I upregulation	(91–93, 96)

### 2.1 ER stress and UPR

**Figure 2:**
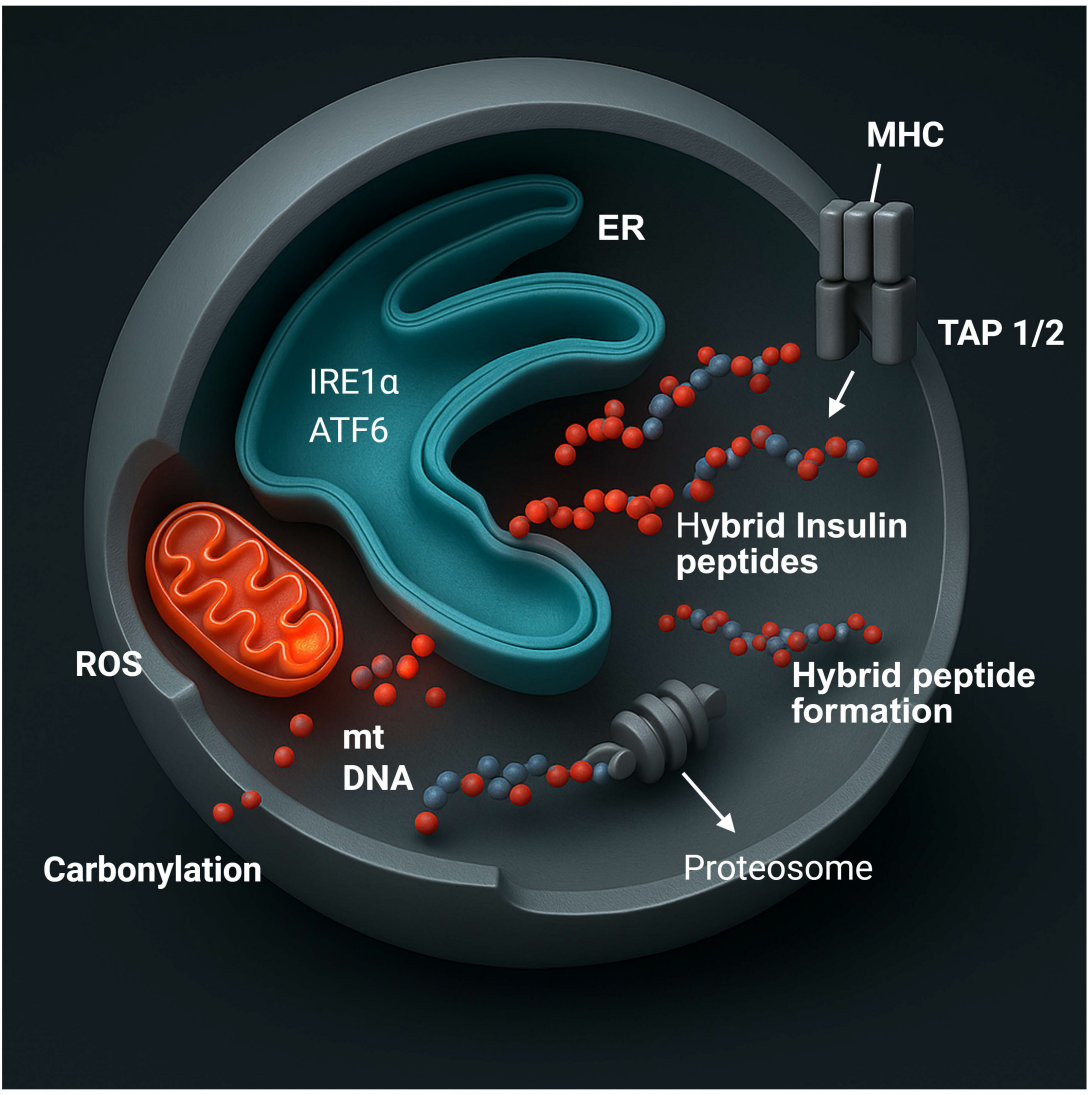
Intracellular integration of β-cell stress pathways in antigen presentation. Metabolic stress within pancreatic β-cells coordinates mitochondrial, endoplasmic-reticulum (ER), and proteasomal responses that enhance antigen visibility. ER stress activates IRE1α and ATF6, increasing peptide-loading capacity and MHC-I expression. Mitochondrial dysfunction produces reactive oxygen species (ROS) and releases mitochondrial DNA (mtDNA), driving oxidative modifications such as protein carbonylation. Proteasomal degradation of oxidized and hybrid insulin peptides yields fragments that are transported into the ER via TAP1/2 for assembly with MHC molecules. The convergence of oxidative stress, organelle crosstalk, and altered peptide processing transforms the β-cell into an active antigen-presenting unit, establishing a mechanistic link between metabolic imbalance and autoimmune activation in type 1 diabetes. Created in BioRender. Mittal, R. (2026) https://BioRender.com/cq8jzkk.

The ER is the site where insulin is synthesized, folded, and assembled before secretion (26–28) (**Figure 2**). During sustained secretory demand or exposure to inflammatory cytokines, misfolded proteins accumulate in the ER lumen and activate the unfolded protein response (29, 30). This adaptive mechanism is mediated by the protein kinase RNA-like ER kinase pathway (PERK), the inositol-requiring enzyme 1 alpha pathway (IRE1α), and the activating transcription factor 6 (ATF6) pathway (26) (**Figure 2**). In β-cells, transient activation of the UPR reduces translation, increases the production of molecular chaperones, and restores homeostasis. Persistent activation, however, induces apoptotic and inflammatory transcriptional programs through CCAAT/enhancer-binding protein homologous protein (CHOP), c-Jun N-terminal kinase (JNK), and nuclear factor kappa B (NF-κB).

These stress pathways influence antigen processing as well as cell survival (24, 25, 31). Splicing of X-box-binding protein 1 (XBP1) enhances transcription of genes that encode peptide-loading components, while the PERK pathway alters global translation and changes the pool of peptides available for binding to MHC-I molecules (32–34). Studies of human and rodent islets show that prolonged ER stress increases the expression of transporter associated with antigen processing 1 and 2 (TAP1/2), β-2-microglobulin, and other elements of the antigen-presentation machinery (35–37). ER stress therefore expands the diversity of peptides processed for MHC-I presentation and sensitizes β-cells to type I IFN, which further increase MHC-I expression and presentation of β-cell–derived antigens (6, 38, 39).

### 2.2 Mitochondrial dysfunction and redox imbalance

Mitochondria are the central regulators of β-cell metabolism as oxidative phosphorylation (OXPHOS) links glucose oxidation to the generation of adenosine triphosphate (ATP) required for insulin exocytosis (40). Impaired mitochondrial respiration increases the production of ROS such as superoxide and hydrogen peroxide (41). In β-cells, these oxidants modify proteins and lipids, generating carbonylated and nitrosylated derivatives that act as novel antigenic determinants (42–44). A persistent oxidative shift in the intracellular redox state alters disulfide bond formation within proinsulin and other secretory proteins, producing misfolded molecules that are processed differently by the proteasome (45–47). These modified peptides enlarge the spectrum of antigens that can be presented on MHC-I molecules (48, 49). Through these mechanisms, mitochondrial ROS directly contribute to β-cell neoantigen generation.

By contrast, mitochondrial damage can also lead to the release of mitochondrial DNA and the phospholipid cardiolipin, which function as danger-associated molecular patterns (DAMPs) once encountered by immune cells. In APCs, these mitochondrial components engage innate immune receptors such as cyclic GMP–AMP synthase (cGAS) and toll-like receptor 9 (TLR-9) (50, 51). Activation of these sensors stimulates type I IFN signaling and the transcription of genes involved in antigen processing (38, 52).

### 2.3 Neoepitope formation through oxidative and redox-dependent mechanisms

**Figure 3:**
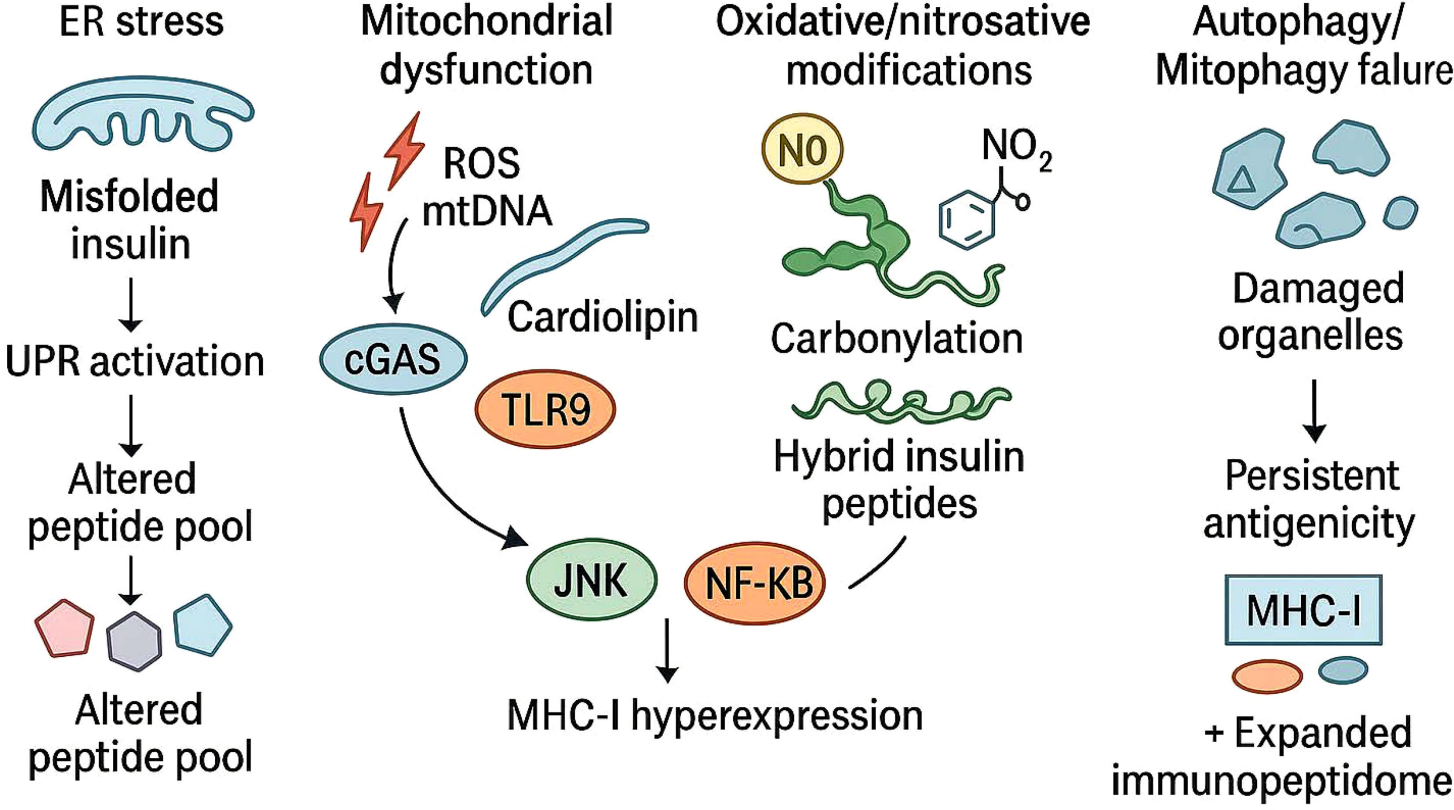
Mechanisms underlying β-cell neoepitope generation. Diverse metabolic and oxidative stress pathways within pancreatic β-cells converge to reshape the antigenic landscape and promote neoepitope formation. Endoplasmic reticulum stress triggers the unfolded protein response (UPR), leading to altered peptide processing and diversification of the MHC class I–associated peptide pool. Mitochondrial dysfunction induces the release of reactive oxygen species (ROS) and mitochondrial DNA (mtDNA), which activate cytosolic and endosomal sensors, including cGAS and TLR9, culminating in MHC-I hyperexpression through JNK and NF-κB signaling. Concurrent oxidative and nitrosative modifications, such as carbonylation, foster the generation of hybrid insulin peptides, whereas impaired autophagy and mitophagy result in the accumulation of damaged organelles and sustained antigen presentation. Collectively, these mechanisms expand the β-cell immunopeptidome and potentiate immune recognition, establishing a molecular basis for autoimmune activation in type 1 diabetes.

One of the most significant consequences of metabolic stress within β-cells is the creation of neoepitopes that are unfamiliar to the immune system (53) (**Figure 3**). Here, β-cell–intrinsic metabolic stress responses reshape the β-cell immunopeptidome by amplifying ER stress, mitochondrial dysfunction, and oxidative injury (54). These perturbations expand the pool of stress-modified peptides and neoepitopes presented on MHC-I molecules, thereby heightening β-cell immunogenicity and promoting early autoimmune recognition (54). Reactive oxygen and nitrogen species alter amino acid side chains, changing charge and conformation of epitopes as well as increasing their affinity for specific HLA alleles (38). Within secretory granules, oxidative conditions promote transpeptidation reactions between fragments of insulin and other granule proteins such as chromogranin A or islet amyloid polypeptide (55). The resulting hybrid insulin peptides (HIPs) are generated within β-cells, displayed at the cell surface, and recognized by autoreactive CD4^+^ T cells (38, 56). However, evidence for CD8^+^ T-cell recognition of HIP-derived epitopes or their presentation by MHC-I remains still unclear.

Extending these observations, recent ultrastructural mapping of the 6.9 HIP using a HIP-specific monoclonal antibody (6.9HIP-MAb) revealed that HIP epitopes localize not only within insulin-positive β-cells but also inside intra-islet antigen-presenting cells in NOD mice (57). Immunogold electron microscopy showed 6.9HIP concentrated within dense-core granules (DCGs) and in granule–lysosomal hybrid compartments marked by LAMP1, indicating that HIP formation or processing occurs within the crinosome/lysosomal axis rather than solely in secretory granules. Notably, induction of ER stress by tunicamycin markedly increased 6.9 HIP abundance in DCG- and crinosome-enriched fractions, establishing a direct mechanistic link between β-cell proteostatic stress and enhanced formation or accumulation of HIP neoantigens (57).

Nitrosative stress contributes further to the diversification of the β-cell immunopeptidome (6, 58, 59). Cytokine-induced expression of inducible nitric oxide synthase (iNOS) leads to production of peroxynitrite, which nitrates tyrosine residues in insulin and other proteins. These nitrated peptides resist degradation by conventional proteasomes and generate unusual cleavage products that enter the MHC pathway (60, 61). The cumulative result of oxidative and nitrosative modification is an expanded set of presented peptides capable of breaking immune tolerance (62–64).

In addition to HIPs, several other biochemical pathways generate neoepitopes during metabolic and inflammatory stress in T1D (10, 65–67) (**Figures 1**, **3**). Carbonylation represents a major oxidative modification that occurs when ROS alter amino acid side chains and convert lysine, arginine, threonine, and proline residues into carbonyl containing groups (68). This highly stable modification changes peptide structure, proteasomal processing, and binding affinity for HLA-I particularly in the presence of mitochondrial dysfunction and elevated ROS (69). Deamidation of asparagine and glutamine residues also contributes to neoepitope formation (67). This reaction increases in acidic and inflamed microenvironments and during prolonged ER stress. Deamidated peptides often display altered charge distribution and can bind HLA-II molecules with increased efficiency.

Citrullination provides another important source of neo-antigens (70, 71). This modification is catalyzed by peptidyl arginine deiminase (PAD) enzymes, which convert arginine into citrulline. PAD activity increases in response to calcium influx, oxidative stress, and exposure to proinflammatory cytokines (72). Citrullinated proteins have been reported in pancreatic islets under inflammatory conditions, and these modified peptides are efficiently processed and presented by APCs that undergo glycolytic metabolic reprogramming (73, 74). Defective ribosomal products (DRiPs) form an additional category of stress-induced neoantigens (75). Activation of the unfolded protein response, (UPR), and the integrated stress response reduces translational fidelity and leads to production of short-lived polypeptides that are rapidly targeted to the proteasome. In cytokine stimulated β-cells and APCs, immunoproteasome induction increases the efficiency with which DRiPs are processed into high affinity ligands for HLA-I molecules.

Together, carbonylation, deamidation, citrullination, and defective ribosomal product generation illustrate how metabolic stress, oxidative injury, and inflammatory signaling reshape the biochemical landscape of antigen formation. These mechanisms progressively widen the diversity of peptides available for presentation and increase the immunological visibility of β-cells. Integrating these established pathways into the broader immunometabolic framework clarifies how microenvironmental cues and cellular metabolism influence both the quality and quantity of neoepitopes that contribute to the initiation and progression of T1D.

### 2.4 Human leukocyte antigen class I hyper-expression and immunopeptidome remodeling

Analyses of pancreatic tissue from individuals in the pre-diabetic and early diabetic stages consistently show pronounced overexpression of human leukocyte antigen (HLA)-I molecules on β-cells (6, 76–79). This phenomenon appears before substantial immune infiltration and may represent an intrinsic response to metabolic and inflammatory stress (77, 80). Type I interferons produced locally activate the Janus-kinase–signal-transducer-and-activator-of-transcription pathway (JAK–STAT) and drive transcription of the HLA-I genes (78, 81, 82). Proteomic mapping of interferon-treated human islets demonstrates both increased surface abundance of class I molecules and a qualitative shift in the presented peptides, with a predominance of HLA-B-restricted epitopes enriched in stress-modified sequences (6, 38, 78, 83). This remodeling enhances recognition of β-cells by cytotoxic lymphocytes and accelerates autoimmune destruction (84–87).

Interferon signaling also induces the formation of immunoproteasomes that generate peptides with C-terminal residues optimized for class I binding (82, 88, 89). The combined induction of immunoproteasomes and peptide-loading machinery ensures efficient presentation of stress-derived antigens and links metabolic perturbation directly to the activation of adaptive immunity (90).

### 2.5 Autophagy and mitophagy in antigen processing

Autophagy is the principal mechanism by which cells remove misfolded proteins and damaged organelles (91–93). In β-cells, autophagy deficiency leads to accumulation of protein aggregates, distension of the ER, and fragmentation of mitochondria (8, 94, 95). Inhibition of autophagy increases ER stress and enhances expression of MHC-I molecules (8, 96, 97). Conversely, stimulation of autophagy restores protein quality control and diminishes antigen presentation (98, 99). The selective clearance of dysfunctional mitochondria through mitophagy prevents the release of mitochondrial DNA and other DAMPs that would otherwise trigger innate immune responses (100–102). Loss of mitophagy regulators such as PINK1 or Parkin in β-cells results in persistent inflammation and amplification of antigenic signals (103–105). Preservation of autophagic flux is therefore essential to limit the immunogenic consequences of metabolic stress (8, 93, 106).

The subsequent section examines how the metabolic state of APCs determines whether these antigens elicit immune tolerance or promote pathogenic immunity, and how continuous dialogue between metabolically stressed β-cells and reprogrammed APCs sustain the autoimmune process in T1D (86, 107) (**Table 1**).

## 3 Immunometabolic regulation of antigen presentation by APCs

APCs represent the crucial bridge between innate and adaptive immunity (**Table 2**). Within the context of T1D, dendritic cells, macrophages, and B cells operate as central mediators that capture β-cell–derived antigens and deliver them to T lymphocytes in pancreatic lymph nodes. These cells do not simply respond to cytokines and antigens; their functional phenotype is determined by their metabolic state. Cellular metabolism in APCs governs the rate and route of antigen uptake, processing, and presentation, and also influences the expression of co-stimulatory molecules that determine the outcome of T-cell activation. The immune and metabolic systems are therefore intertwined at a fundamental level. Understanding this relationship provides new insight into how metabolic cues within the pancreatic microenvironment shape the evolution of autoimmunity.

**Table 2:** Immunometabolic regulation of antigen presentation in Antigen-Presenting Cells (APCs).

Metabolic Pathway/regulator	Functional outcome	Antigen-Presentation effect	Key references
mTORC1 Activation	Promotes glycolysis and IL-12 production	Enhances pro-inflammatory antigen presentation	(16, 17, 127, 237)
AMPK Activation	Increases oxidative metabolism, mitochondrial biogenesis	Induces tolerogenic phenotype with IL-10 secretion	(18, 304)
GCN2/Integrated Stress Response	Senses amino acid deprivation	Alters translation and enhances antigen-processing gene expression	(128–130, 243, 244)
Lipid Metabolism Alterations	Lipid droplet accumulation; oxidized lipid uptake	Impairs peptide-MHC loading, enhances CD1-mediated lipid antigen presentation	(131–136)
Mitochondrial ROS Production	Moderate ROS enhances cross-presentation; excessive ROS causes peptide damage	Fine-tunes balance between tolerance and inflammation	(152–154)
Hypoxia/HIF-1α Stabilization	Shifts metabolism toward glycolysis; increases iNOS and cytokine production	Favors inflammatory antigen presentation	(23, 98, 110, 173, 176)

### 3.1 The islet microenvironment and the metabolic landscape of APCs

The pancreatic islet is a metabolically active micro-organ in which insulin secretion and nutrient flux generate substantial variation in oxygen tension, glucose concentration, and lipid content (108–110). Islet-resident macrophages and dendritic cells are continuously exposed to this changing environment (86, 111). During the initial stages of insulitis, infiltrating immune cells and stressed β-cells release cytokines, reactive oxygen species (ROS), and metabolic by-products that remodel the local milieu. Hypoxia, nutrient deprivation, and accumulation of lactate and fatty acids alter the bioenergetic programs of islet macrophages (23, 108, 110).

Under basal conditions, islet macrophages rely on OXPHOS to maintain tissue homeostasis and phagocytic clearance of apoptotic β-cells without eliciting inflammation (112–115). In contrast, during inflammation, activation of TLRs or exposure to cytokines such as IFN-γ induces a metabolic switch toward glycolysis (116). This glycolytic reprogramming supports pro-inflammatory mediator synthesis and enhances MHC-II–restricted presentation of captured β-cell antigens, together with increased co-stimulation (117). Single-cell transcriptomic analyses of human and murine islets confirm that the shift from oxidative to glycolytic metabolism is accompanied by increased expression of MHC-II molecules, co-stimulatory ligands, and cytokines that promote T-cell activation (25, 118).

### 3.2 Nutrient-sensing and stress-responsive signaling in APCs

Nutrient-sensing pathways function as molecular rheostats that adjust the immune capacity of APCs to their metabolic environment (119, 120). The mechanistic target of rapamycin complex 1 integrates signals from amino acids, glucose, and growth factors to promote anabolic metabolism and pro-inflammatory activity (119, 121–123). Activation of this pathway enhances glycolytic flux and supports the production of IL-12 and other cytokines that favor T helper type 1 polarization (120, 123–125). In contrast, activation of AMPK favors oxidative metabolism, mitochondrial biogenesis, and anti-inflammatory or tolerogenic phenotypes (126, 127).

Another critical regulator is the general control non-derepressible 2 kinase (GCN2), which detects amino acid deprivation and triggers the integrated stress response (128–130). Engagement of this pathway modifies mRNA translation and activates transcription factors such as activating transcription factor 4 (ATF-4), which can influence antigen-processing genes (129, 130). When nutrients are scarce, activation of this stress response may paradoxically increase antigen presentation while impairing the induction of regulatory signals, thereby promoting autoreactivity. Collectively, these nutrient sensors coordinate metabolic activity with immune function and determine whether an APC promotes tolerance or immunity.

### 3.3 Influence of lipid metabolism on antigen processing

Lipid metabolism exerts profound effects on antigen presentation (131–134). In APCs, accumulation of intracellular lipid droplets interferes with endosomal trafficking and the assembly of peptide–MHC complexes (15, 135–138). In metabolic disorders characterized by hyperlipidemia or obesity, this phenomenon contributes to immune dysfunction (132, 134, 139). Within the pancreatic islet, elevated levels of free fatty acids and oxidized lipids derived from stressed β-cells can modify the lipid composition of macrophage membranes and endosomes (140, 141). These alterations may impair the processing of conventional peptide antigens while enhancing the presentation of lipid antigens through CD1 molecules (133, 142–144). Lipid-reactive natural killer T cells, which recognize glycolipid antigens, are increasingly implicated in the regulation of autoimmunity and may play an underappreciated role in T1D (145, 146).

Lipid peroxidation products such as 4-hydroxynonenal (4-HNE) can form covalent adducts with proteins within APCs, generating modified self-molecules that act as adjuvants or new epitopes (147–149). These products also activate nuclear receptors such as peroxisome proliferator-activated receptors (PPARγ), which in turn modulate gene expression related to metabolism and antigen presentation (150, 151). The integration of lipid metabolic signals with immune signaling thus provides another axis of regulation that determines the immunogenic potential of islet APCs.

### 3.4 Mitochondrial function and redox signaling in APC activation

Mitochondria in APCs serve not only as energy generators but also as regulators of redox balance and innate immune signaling (152–154). Whereas MHC-II presentation is typically increased during inflammatory glycolytic activation, mitochondrial ROS signaling can be particularly important for cross-presentation of exogenous β-cell antigens on MHC-I. Controlled production of mitochondrial ROS is necessary for efficient antigen cross-presentation because low-level oxidative bursts promote the release of endosomal antigens into the cytosol where they are processed for MHC-I presentation (155, 156). Excessive mitochondrial oxidative stress, however, damages peptides and impairs peptide loading onto MHC molecules (156–160).

Mitochondrial metabolism also contributes to the generation of metabolites such as succinate and fumarate, which function as signaling molecules (161–164). Succinate accumulation stabilizes hypoxia-inducible factor 1 alpha (HIF1α), thereby enhancing glycolytic metabolism and promoting the expression of pro-inflammatory genes (165–168). In contrast, intact tricarboxylic acid cycle (TCA) activity and efficient OXPHOS are associated with anti-inflammatory and tolerogenic functions (169, 170). The balance between these metabolic programs influences whether the APC induces effector T-cell responses or regulatory T-cell differentiation (171).

### 3.5 Hypoxia, lactate accumulation, and the metabolic polarization of APCs

Inflamed pancreatic islets often experience local hypoxia because of immune cell infiltration and vascular disruption (23, 110, 172). Hypoxia stabilizes HIF1α, a transcription factor that promotes glycolytic metabolism and enhances the expression of iNOS and co-stimulatory molecules (15, 98, 173). Hypoxia-induced metabolic polarization supports antigen presentation and increases the production of cytokines such as TNF and IL-1 β, both of which intensify β-cell stress (98, 137).

Simultaneously, hypoxic metabolism increases the generation of lactate, which has context-dependent effects on immune cells (174–178). In tolerogenic dendritic cells, lactate accumulation can inhibit nuclear factor kappa B (NF-κB) activation and reduce co-stimulatory molecule expression, whereas in pro-inflammatory settings, lactate serves as an additional substrate for histone acetylation, promoting transcription of inflammatory genes (176, 177, 179). The net effect in the diabetic islet is complex but tends toward inflammation because of the concurrent presence of interferons and cytokines that dominate over tolerogenic influences (180).

## 4 Reciprocal crosstalk between β-cells and antigen-presenting cells

**Figure 4:**
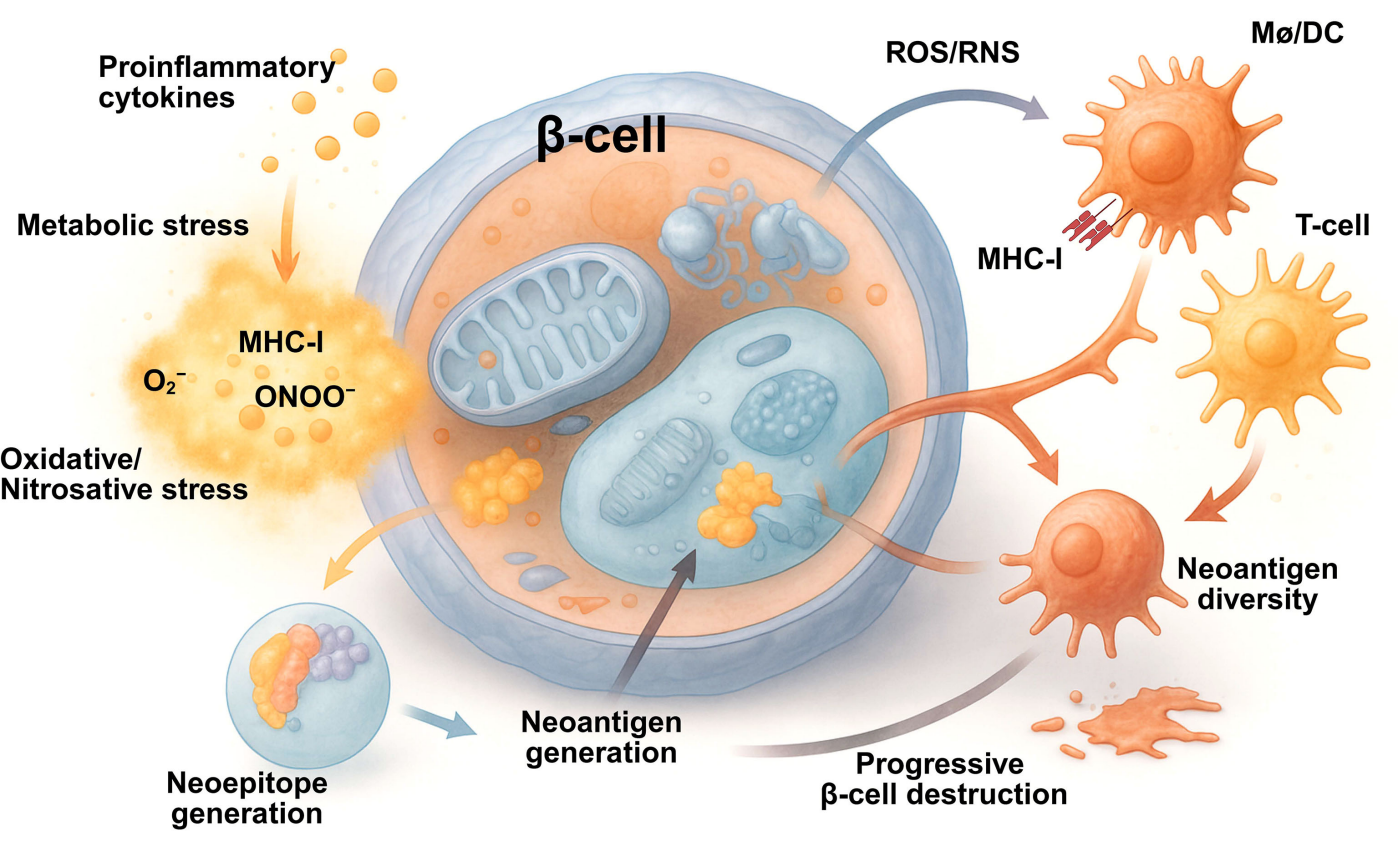
Immunometabolic remodeling of β cells drives neoantigen generation and autoimmune amplification in type 1 diabetes. Metabolic stress in pancreatic β cells induced by proinflammatory cytokines nutrient imbalance and mitochondrial dysfunction promotes oxidative and nitrosative stress with increased reactive oxygen and nitrogen species. These perturbations disrupt proteostasis and redox homeostasis leading to post translational modifications defective protein processing and neoepitope formation. Stressed β cells upregulate major histocompatibility complex (MHC)-I enhancing presentation of modified self-peptides. Neoantigens released from stressed or dying β cells are captured by antigen presenting cells that undergo metabolic and redox reprogramming to enhance antigen processing and presentation. Activation of autoreactive T cells establishes a feed forward inflammatory loop that amplifies immune responses and accelerates progressive β cell destruction linking immunometabolic stress to loss of immune tolerance in type 1 diabetes. Created in BioRender. Mittal, R. (2026) https://BioRender.com/9sr96th.

The interaction between pancreatic β-cells and APCs forms a central pathogenic circuit in T1D. The two cell types engage in continuous bidirectional communication through soluble mediators, metabolic signals, and direct cell-to-cell contact (**Table 3**). Metabolic stress in β-cells alters the repertoire of antigens that are released and modifies the extracellular environment, whereas APCs respond to these cues by changing their metabolic state and immune behavior. This reciprocal communication amplifies inflammation and accelerates the breakdown of immune tolerance (**Table 3**; **Figure 4**).

**Table 3:** Reciprocal crosstalk between β-Cells and Antigen-Presenting Cells (APCs) in Type 1 diabetes.

Interaction axis	Molecular mediators	Pathophysiological effect	Key references
β-Cell → APC Communication	Release of mtDNA, HSPs, HMGB1, ATP, oxidized peptides	Activation of TLRs and cGAS–STING in APCs; cytokine release	(49, 78, 181, 197, 198)
APC → β-Cell Feedback	IFN-α/β, TNF, IL-1β, IL-12	Induces ER and oxidative stress; upregulates MHC-I	(25, 90, 305, 306)
Nutrient and Oxygen Competition	Hypoxia, amino acid limitation, glucose depletion	HIF-1α and GCN2 activation; amplifies inflammation	(110, 189–195)
Cytokine Loop Reinforcement	Persistent IFN and TNF signaling	Chronic antigen presentation and T-cell activation	(35, 36, 38, 127)

### 4.1 Release of danger signals and modified antigens from stressed β-cells

Under metabolic or oxidative stress, β-cells release a variety of danger-associated molecular patterns, including heat-shock proteins, mitochondrial DNA, high-mobility group box 1 protein (HMGB1), and extracellular ATP (78, 181, 182) (**Figure 4**). These molecules activate pattern-recognition receptors (PRRs) on macrophages and dendritic cells, such as TLRs and the nucleotide-binding oligomerization domain-like receptor family (NLR), leading to the production of inflammatory cytokines. Simultaneously, apoptotic and necrotic β-cells release intracellular proteins that have undergone oxidative or nitrosative modification. These altered proteins serve as a rich source of potential autoantigens. Once taken up by APCs, they are processed and loaded onto MHC-II molecules (with cross-presentation onto MHC-I in appropriate APC subsets) for presentation to autoreactive T lymphocytes.

The presence of ROS within the islet microenvironment not only modifies proteins but also oxidizes extracellular lipids and nucleic acids, which can further stimulate innate immune receptors. This combination of danger signals and modified self-antigens transforms the islet from a quiescent metabolic organ into a site of persistent immune activation.

### 4.2 Antigen uptake, processing, and metabolic activation of APCs

APCs in the islet and in the draining pancreatic lymph node internalize β-cell-derived material through phagocytosis, receptor-mediated endocytosis, and micropinocytosis (183). The metabolic state of these cells influences how efficiently they process these antigens. Inflammatory cytokines, such as interferon gamma, promote glycolytic reprogramming, which enhances the expression of proteasome components, peptide transporters, and co-stimulatory molecules (6). This metabolic activation increases the likelihood that β-cell antigens will be presented in a highly immunogenic context. In this setting, APC activation primarily augments MHC-II presentation of β-cell antigens, while also increasing the efficiency of cross-presentation on MHC-I.

Conversely, when OXPHOS predominates and nutrients are scarce, APCs exhibit a more tolerogenic profile characterized by limited co-stimulation and secretion of anti-inflammatory mediators (173). In T1D, however, chronic exposure to cytokines, hypoxia, and high levels of free fatty acids skews the metabolic balance toward glycolysis and sustains inflammatory antigen presentation (23). The result is a persistent population of activated dendritic cells and macrophages that maintain autoreactive T-cell stimulation (111).

### 4.3 Metabolic coupling and nutrient competition

The inflamed islet represents a metabolically competitive environment (184, 185). Infiltrating immune cells consume glucose and amino acids, reducing nutrient availability for neighboring β-cells (122, 186, 187). This deprivation triggers integrated stress responses in both cell types (188). In β-cells, reduced glucose uptake suppresses insulin biosynthesis but increases oxidative stress through mitochondrial inefficiency (110, 189–191). In APCs, nutrient scarcity activates the GCN2 and shifts translation toward stress-responsive genes that support antigen presentation and cytokine production (192–195).

Competition for oxygen further exacerbates this relationship (23, 184, 196). As inflammation progresses, vascular perfusion becomes impaired, generating local hypoxia that stabilizes HIF1α in macrophages and dendritic cells (122, 179, 184). This stabilization drives glycolytic metabolism and pro-inflammatory gene expression (122, 173). The same hypoxic environment damages β-cell mitochondria, enhancing ROS formation and the release of mitochondrial components that serve as innate immune triggers (197, 198). Thus, metabolic coupling through shared resources and microenvironmental changes reinforces pathological communication between the two cell populations (184, 185).

## 5 Translational perspectives and therapeutic implications

**Figure 5:**
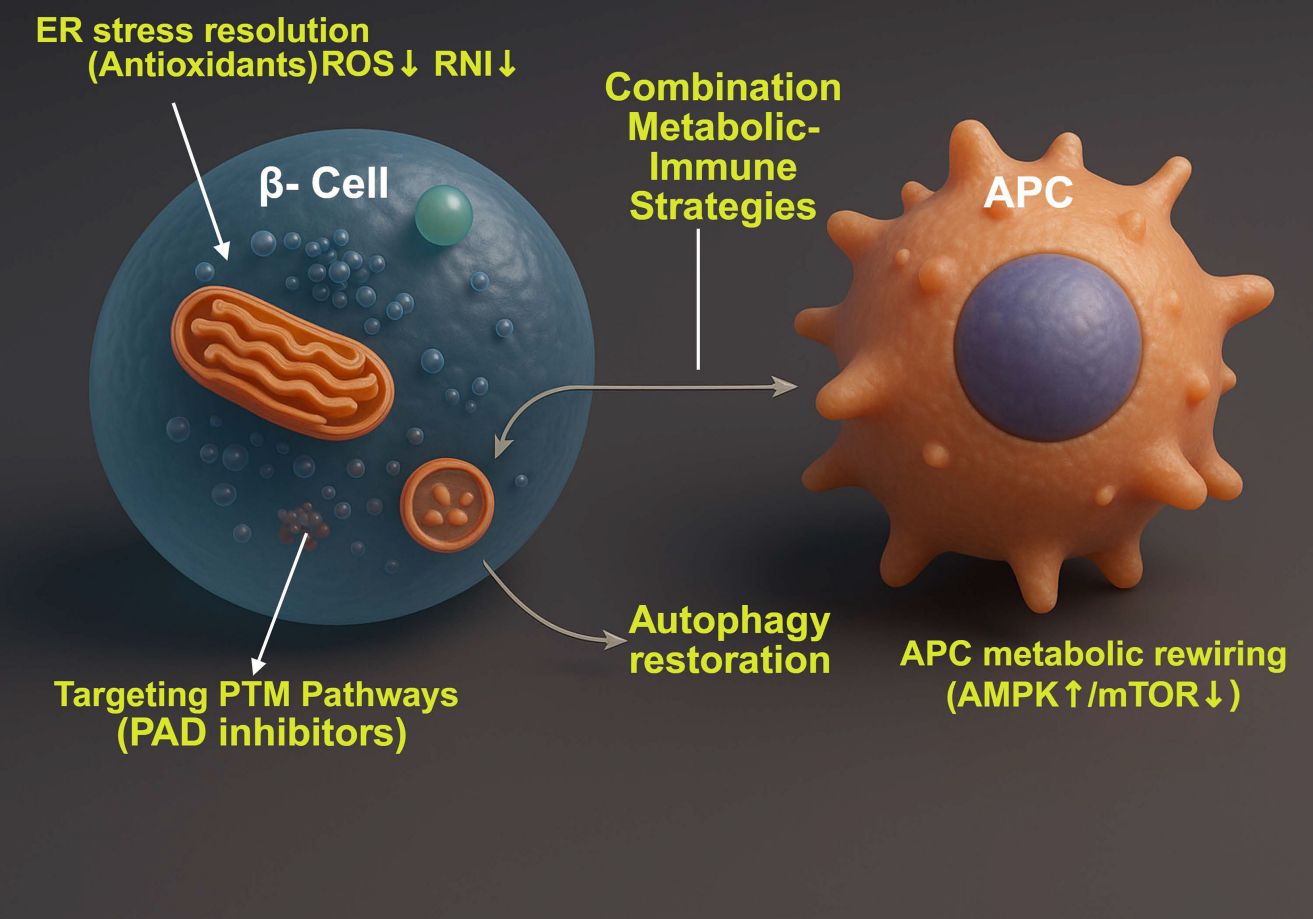
Immunometabolic targeting of the β-cell–APC axis in type 1 diabetes. Metabolic and oxidative stress in β-cells induces ER stress, redox imbalance, post-translational modifications, impaired autophagy, and neoepitope generation, enhancing antigenicity. Interventions that reduce ROS/RNS, inhibit PTM pathways (e.g., PAD inhibition), and restore autophagy stabilize β-cell proteostasis and limit antigen production. Concurrently, APCs undergo inflammatory metabolic reprogramming characterized by increased mTOR signaling and glycolysis. Rewiring APC metabolism toward AMPK activation and mTOR suppression promotes tolerogenic antigen processing and presentation. Integrated β-cell– and APC-directed immunometabolic strategies may disrupt feed-forward autoimmunity and restore immune tolerance in type 1 diabetes. Created in BioRender. Mittal, R. (2026) https://BioRender.com/j8d9or0.

Understanding the metabolic relationship between pancreatic β-cells and APCs reveals new therapeutic opportunities (**Table 4**). Traditional strategies for T1D have focused primarily on immune suppression or antigen-specific tolerance induction. Although these approaches can delay disease progression, they rarely prevent relapse as the underlying metabolic stress that drives antigen generation and presentation remains uncorrected. A complementary strategy aims to restore metabolic homeostasis within both β-cells and APCs, thereby reducing antigenicity and dampening immune activation at its source (**Figure 5**).

**Table 4:** Therapeutic strategies targeting the immunometabolic interface.

Target/Strategy	Mechanism	Expected outcome	Supporting evidence
ER Stress Modulators (e.g., TUDCA, 4-PBA)	Chemical chaperones stabilize protein folding	Restore β-cell proteostasis; reduce MHC-I upregulation	(200–202)
Antioxidants (e.g., MitoQ, SkQ1)	Scavenge mitochondrial ROS	Reduce oxidative neoantigens, preserve insulin secretion	(204–213)
Autophagy Enhancers (e.g., Trehalose, Spermidine)	Promote clearance of damaged organelles	Limit antigen release and inflammation	(211–213)
mTORC1 Inhibitors/AMPK Activators (e.g., Rapamycin, Metformin)	Rewire APC metabolism	Promote tolerogenic phenotype; suppress IL-12/TNF	(18, 232–237)
Metabolic Combination Therapy	Dual targeting of β-cell and APC metabolism	Break β-cell–APC feedback loop	(8, 38)

### 5.1 Restoring β-cell proteostasis and metabolic stability

β-cell stress is the initial spark that ignites immune recognition (8). Interventions that preserve protein-folding capacity, reduce oxidative injury, or enhance organelle quality control can attenuate this process (199). Small-molecule chaperones such as tauroursodeoxycholic acid and 4-phenylbutyric acid assist in the resolution of ER stress by stabilizing protein conformation and facilitating proper folding (200–202). Experimental studies in rodent models demonstrate that these agents reduce expression of inflammatory genes, normalize insulin synthesis, and protect β-cell mass (199, 203). Pharmacologic activators of nuclear factor erythroid 2–related factor 2 (Nrf2) enhance the antioxidant defense system, decreasing ROS formation and preventing the oxidative modifications that give rise to neoantigens (44, 199).

Mitochondria-targeted antioxidants such as MitoQ and SkQ1 scavenge ROS directly within the mitochondrial matrix (204–206). Their use in preclinical models lowers lipid peroxidation, reduces cytokine production, and maintains insulin secretion (207–210). Agents that stimulate autophagy, including trehalose and spermidine, promote clearance of misfolded proteins and damaged mitochondria, restoring proteostasis and limiting the accumulation of potentially immunogenic debris (211–213). Collectively, these interventions reinforce the natural adaptive stress responses of the β–cell and decrease its visibility to the immune system.

### 5.2 Targeting PTM pathways to limit neoantigen generation

In addition to therapeutic strategies aimed at mitigating oxidative stress and ER stress, direct targeting of PTM pathways that contribute to neoantigen formation represents a complementary approach for modifying the progression of autoimmune diabetes (214–217). PTMs alter the biochemical and structural properties of β-cell proteins, generating antigenic determinants that are not encountered during thymic selection and therefore evading central tolerance (218–220). As a result, these modified epitopes exhibit an increased likelihood of recognition by autoreactive T cells (221, 222). Among the PTMs implicated in β-cell autoimmunity, protein citrullination has emerged as a particularly significant source of β-cell neoepitopes (217, 219, 221).

Citrullination is mediated by PAD enzymes, whose activity is regulated by intracellular calcium flux and is enhanced under conditions of metabolic stress and inflammatory signaling (217, 223). In β-cells, these stress associated cues promote the conversion of arginine residues to citrulline within key autoantigens, leading to altered peptide processing, modified MHC binding properties, and changes in TCR engagement (219, 221). The resulting citrullinated peptides expand the β-cell immunopeptidome and increase the probability of immune recognition by autoreactive lymphocytes.

Pharmacologic inhibition of PAD activity has provided direct experimental evidence supporting a pathogenic role for citrullination in autoimmune diabetes (214). In the NOD mouse model, PAD inhibition reduces the accumulation of citrullinated β-cell proteins, limits the availability of modified antigenic peptides, and attenuates autoreactive T cell activation (214, 224). These immunologic effects are associated with delayed disease onset and a reduced incidence of diabetes, demonstrating that suppression of neoantigen generation can produce meaningful disease modification *in vivo*.

Targeting PTM pathways addresses a distinct pathogenic axis that operates upstream of immune effector activation (214, 224). Rather than broadly suppressing inflammatory signaling or adaptive immune responses, PAD inhibition selectively limits the formation of highly immunogenic antigens while preserving basal immune competence. This antigen focused strategy complements approaches aimed at modulating β-cell stress responses or immune cell metabolic programs and highlights neoantigen generation as a mechanistically defined and therapeutically amenable target. Collectively, these findings support the concept that limiting the production of stress induced neoantigens at their cellular source can reshape immune recognition and alter the trajectory of autoimmune diabetes. Therapeutic strategies that constrain PTM driven antigen diversification may therefore contribute to the preservation of immune tolerance and long-term β-cell function, particularly when implemented during early or pre symptomatic stages of disease (214, 224).

### 5.3 Modulating β-cell redox and signaling networks

Control of the cellular redox environment provides an additional layer of therapeutic leverage. Redox-active signaling regulates not only oxidative defense but also gene transcription, calcium homeostasis, and apoptosis (225, 226). Modest activation of redox-sensitive pathways through physiological stressors such as intermittent fasting or mild caloric restriction induces adaptive hormesis, enhancing cellular resilience (227). Conversely, chronic oxidative overload triggers maladaptive signaling that promotes inflammation and antigen presentation (228). Pharmacologic manipulation of the glutathione and thioredoxin systems, as well as supplementation with precursors of nicotinamide adenine dinucleotide, can restore a balanced redox tone (229).

The regulation of cytokine receptor signaling also intersects with metabolic pathways. Inhibition of the JAK–STAT axis reduces interferon-driven expression of MHC-I molecules and lowers antigenic load (230). Such approaches must be applied judiciously to avoid generalized immune suppression, but transient modulation during early disease stages may shift the balance toward tolerance (231).

### 5.4 Reprogramming antigen-presenting cell metabolism

Parallel to the protection of β-cells, therapeutic benefit may be achieved by redirecting the metabolic programming of APCs (18, 232–234). The mechanistic target of rapamycin complex 1 is a key driver of glycolytic, pro-inflammatory metabolism (235–237). Inhibition of this pathway through rapamycin analogs or caloric restriction enhances OXPHOS and promotes a tolerogenic phenotype characterized by reduced production of IL-12 and TNF (238–240). In contrast, activation of AMPK increases mitochondrial biogenesis and drives expression of genes associated with anti-inflammatory cytokines such as interleukin 10 (18, 241, 242).

Other interventions exploit metabolic checkpoints associated with amino acid sensing (243–245). Activation of the GCN2 pathway by limited amino acid availability suppresses translation of inflammatory mediators and can promote immune tolerance if properly controlled (246–248). Supplementation with specific metabolites such as tryptophan or arginine can further modulate immune function through their roles in regulatory pathways and nitric oxide synthesis (249–251).

Lipid metabolism also provides an avenue for intervention (252–254). The PPARγ agonists regulate lipid uptake and storage and have been shown to reduce inflammation in macrophages (255, 256). By normalizing lipid metabolism within APCs, these agents may restore the balance between antigen clearance and presentation (18, 257).

### 5.5 Combination strategies and timing of intervention

The immunometabolic feedback loop linking β-cells and APCs suggests that effective therapy will likely require simultaneous modulation of both cell types (8, 38). Combination approaches could pair an ER chaperone or mitochondrial antioxidant with an agent that redirects APC metabolism toward tolerance. This dual modulation would both decrease antigen generation and reduce immune responsiveness to remaining antigens (18).

The earliest stages of T1D, when autoantibodies appear but insulin secretion remains measurable, offer the greatest potential for intervention as timing is critical (258, 259). At this stage, β-cell stress is still reversible, and the immune system retains plasticity. Clinical monitoring tools such as proinsulin-to-C-peptide ratio, circulating interferon-stimulated gene expression, and metabolic imaging can help identify individuals in this window (260, 261). Intervening before extensive β-cell destruction occurs may facilitate in slowing or halting the disease process (262).

### 5.6 Technological advances enabling metabolic therapy

Emerging technologies facilitate the translation of these concepts into clinical application. Single-cell transcriptomic and metabolomic analyses allow detailed mapping of metabolic states in β-cells and immune cells from human donors (263–265). Real-time metabolic flux assays and noninvasive imaging techniques can monitor the effects of therapy on cellular bioenergetics (264, 266–270). The integration of these tools with computational modeling will permit precise adjustment of therapeutic regimens to individual metabolic profiles.

Advances in nanotechnology and biomaterials further enhance the specificity of metabolic interventions. Nanoparticles can deliver antioxidants, small interfering RNA, or metabolic modulators directly to pancreatic islets, minimizing systemic side effects (271, 272). Encapsulation of β-cells in biomaterials engineered to modulate local oxygen and nutrient gradients offers another route to protect transplanted or regenerating cells from immune attack (273, 274).

## 6 Biomarkers and future research directions

The elucidation of metabolic and immune interactions in T1D has created new opportunities for identifying biomarkers that reflect the earliest pathogenic changes in both β-cells and APCs (**Table 5**). These biomarkers can serve as indicators of disease risk, guides for therapeutic intervention, and quantitative measures of treatment efficacy. The integration of metabolic, immunologic, and molecular readouts offers a comprehensive approach to disease monitoring and provides a foundation for precision medicine.

**Table 5:** Emerging biomarkers of immunometabolic dysfunction in type 1 diabetes.

Biomarker category	Example(s)	Clinical/Research relevance	References
β-Cell Stress Markers	Proinsulin:C-peptide ratio, GRP78, CHOP expression	Reflects ER stress and impaired insulin processing	(275–277)
Damage-Associated Molecular Patterns (DAMPs)	Circulating mtDNA, oxidized peptides	Correlates with interferon gene signatures	(260, 261)
Oxidative Stress Indicators	Plasma ROS, antioxidant enzyme activity	Quantifies systemic redox imbalance	(285)
Immunopeptidomic Signatures	Hybrid insulin peptides, HLA-B–restricted neoepitopes	Track β-cell immunogenic remodeling	(6, 42, 56)
APC Metabolic Profiles	Lactate/citrate ratio, succinate accumulation, AMPK activity	Indicates pro-inflammatory vs. tolerogenic state	(286–288)

### 6.1 Biomarkers of β-cell stress and antigenicity

β-cell stress is characterized by a constellation of molecular and biochemical alterations that can be measured in blood or in isolated islets (25, 260). Elevated circulating proinsulin relative to C-peptide is a well-validated indicator of impaired proinsulin processing and ER dysfunction (275–277). This ratio increases months to years before the onset of hyperglycemia and therefore serves as an early warning of β-cell stress. Additional biomarkers include the release of ER chaperones such as glucose-regulated protein 78 (GRP78), the up-regulation of CHOP in islet tissue, and the detection of fragments of insulin or proinsulin containing oxidative modifications (24, 278, 279).

Circulating cell-free mitochondrial DNA provides another readout of metabolic distress (280, 281). Elevated levels of mitochondrial DNA are associated with the release of DAMPs and correlate with IFN-stimulated gene signatures in peripheral blood (282–284). Measurements of ROS and antioxidant enzyme activity in plasma also provide indirect evidence of systemic oxidative stress (285). Together, these markers capture distinct aspects of the β-cell response to metabolic challenge and can be used to monitor the impact of therapies aimed at restoring cellular homeostasis.

### 6.2 Immunopeptidomic profiling and detection of neoantigens

Advances in mass spectrometry have enabled the direct characterization of the peptides bound to HLA molecules in islet tissue and in peripheral blood mononuclear cells (6, 42). Immunopeptidomic profiling can identify stress-induced peptides, HIPs, and oxidatively modified epitopes that arise during disease progression. Quantitative changes in the abundance or composition of these peptides can serve as molecular fingerprints of β-cell immunogenicity.

High-resolution mass spectrometric analysis can also detect differences in peptide presentation associated with specific HLA alleles, including the skewing toward HLA-B–restricted peptides that occurs after interferon exposure. Monitoring these changes in at-risk individuals may allow early identification of those transitioning from immune tolerance to active autoimmunity. The detection of circulating immune complexes containing neoantigenic peptides could further refine prediction of disease onset.

### 6.3 Biomarkers of APC metabolic state and antigen-presenting function

The metabolic polarization of APCs can be assessed through transcriptomic and metabolomic analyses of peripheral blood monocytes and dendritic cells (286–288). Expression patterns of genes involved in glycolysis, OXPHOS, and lipid metabolism reveal the predominant metabolic program (286, 289). Ratios of glycolytic to oxidative metabolites, such as lactate to citrate or succinate to fumarate, provide quantitative indicators of metabolic activity (167, 290, 291).

Flow cytometric analysis of co-stimulatory molecule expression combined with measurement of intracellular ATP and mitochondrial membrane potential allows direct assessment of APC activation (292–294). Single-cell RNA sequencing integrated with metabolite mapping can uncover subsets of APCs with distinct immunometabolic profiles that correlate with disease progression (265, 295, 296). These cellular and molecular signatures can be used to evaluate whether therapeutic interventions successfully reprogram APC metabolism toward a tolerogenic phenotype.

### 6.4 Integration of multi-omic biomarkers for personalized monitoring

The complexity of the immunometabolic network in T1D necessitates the integration of multiple layers of data. Multi-omic approaches that combine genomics, transcriptomics, proteomics, metabolomics, and immunopeptidomics provide a systems-level understanding of disease dynamics (297, 298). Machine-learning algorithms can identify biomarker combinations that predict progression from the preclinical stage to overt diabetes with high accuracy (299).

Longitudinal sampling of at-risk individuals can reveal temporal patterns of β-cell stress and immune activation, allowing the establishment of dynamic disease trajectories (300, 301). These trajectories can then guide the timing and intensity of therapeutic interventions. The use of standardized sample-collection protocols and analytical platforms will be essential to ensure reproducibility and comparability across studies.

### 6.5 Conceptual implications and outlook

The convergence of metabolism and immunity in the pathogenesis of T1D transforms the conceptual framework of the disease (302). It suggests that autoimmune destruction results not only from immune dysregulation but also from the failure of metabolic adaptation within the target tissue (303). This insight positions the β-cell as an active participant in disease initiation rather than a passive casualty.

Future progress will depend on the close collaboration between immunologists, metabolic biologists, and systems scientists. Integrating their perspectives will enable the construction of comprehensive models that capture the dynamic interplay among stress signaling, antigen presentation, and immune recognition. Such models will provide the basis for predictive diagnostics and rational therapeutic design.

In this view, successful management or prevention of T1D will rely on restoring cellular homeostasis rather than simply suppressing immune reactivity. Interventions that preserve the metabolic integrity of β-cells and recalibrate the energy metabolism of APCs hold the promise of transforming T1D from an inexorable autoimmune process into a controllable, and possibly reversible, metabolic disorder.

## 7 Concluding remarks

The integration of metabolic and immunologic research has fundamentally changed our understanding of T1D. The disease is no longer viewed solely as an autoimmune assault mediated by misdirected lymphocytes but rather as a complex disorder of cellular communication in which metabolic dysfunction initiates and sustains immune activation. Within this conceptual framework, β-cell and APCs emerge as equal partners in a pathological dialogue that drives the loss of immune tolerance and the destruction of pancreatic islets.

The evidence now supports the existence of a continuous immunometabolic loop in which stress within the β-cell and metabolic activation of APCs reinforce each other. In the β-cell, ER stress, mitochondrial dysfunction, and oxidative imbalance give rise to an altered immunopeptidome enriched in modified or hybrid peptides (10). These changes increase antigen visibility and provoke innate immune responses. At the same time, APCs exposed to inflammatory cytokines, hypoxia, and nutrient fluctuations adopt metabolic programs that favor glycolysis, production of ROS, and expression of co-stimulatory molecules (23). This metabolic polarization enhances antigen processing and cross-presentation, perpetuating autoreactive T-cell activation.

The persistent exchange of metabolic and inflammatory signals between these two cellular populations transforms the islet microenvironment into a self-sustaining immune niche. Cytokines such as IFN, TNF, and IL-1β amplify oxidative and ER stress, while reactive oxygen and nitrogen species propagate tissue damage and generate further neoantigens. The resulting feedback circuit not only accelerates β-cell loss but also stabilizes immune memory against self-antigens, making the autoimmune process resistant to conventional immunosuppression.

Recognition of this immunometabolic loop has far-reaching therapeutic implications. Interventions that restore metabolic equilibrium within the β-cell, reinforce antioxidant defenses, and preserve proteostasis can limit antigen generation at its origin. Likewise, modulation of APC metabolism through activation of adenosine monophosphate–activated protein kinase or inhibition of mechanistic target of rapamycin can re-establish a tolerogenic phenotype that restrains autoreactive T-cell activation. When these metabolic interventions are combined with antigen-specific immunotherapy, they offer the potential to reset the immune system without compromising host defense.

The conceptual shift from immune suppression to metabolic rehabilitation reframes the ultimate goal of therapy. The objective is no longer to silence the immune system but to reconstitute the physiological dialogue between immune cells and metabolic tissues. This approach envisions T1D diabetes as a reversible imbalance of cellular homeostasis that can be corrected by synchronizing metabolic and immune pathways.

Future research should pursue three interconnected goals. The first is to delineate the molecular mechanisms through which redox signaling and protein modification alter antigen presentation. The second is to identify biomarkers that accurately reflect immunometabolic states *in vivo* and that can guide early intervention. The third is to develop therapeutic combinations that simultaneously stabilize β-cell metabolism and recalibrate immune-cell energy utilization. Success in these areas will move the field from observation to intervention, transforming mechanistic insights into clinical benefit.

In summary, metabolic stress in β-cells and APCs constitutes a unifying axis that explains the initiation and propagation of autoimmune injury in T1D. The interactions between these cell types are mediated through shared metabolic pathways, redox signaling, and antigen-processing mechanisms that form a continuous and self-reinforcing circuit. Targeting this circuit represents a powerful strategy to preserve β-cell mass, restore immune tolerance, and ultimately alter the natural history of the disease. The synthesis of immunology and metabolism thus defines a new paradigm for understanding and treating T1D and may provide a blueprint for addressing other chronic autoimmune disorders rooted in metabolic dysfunction.
